# Attention and working memory deficits in a perinatal nicotine exposure mouse model

**DOI:** 10.1371/journal.pone.0198064

**Published:** 2018-05-24

**Authors:** Lin Zhang, Thomas J. Spencer, Joseph Biederman, Pradeep G. Bhide

**Affiliations:** 1 Center for Brain Repair, Biomedical Sciences, Florida State University College of Medicine, Tallahassee, United States of America; 2 Pediatric Psychopharmacology, Department of Psychiatry, Massachusetts General Hospital, Harvard Medical School, Boston, United States of America; University of Missouri Columbia, UNITED STATES

## Abstract

**Background:**

Cigarette smoking by pregnant women is associated with a significant increase in the risk for cognitive disorders in their children. Preclinical models confirm this risk by showing that exposure of the developing brain to nicotine produces adverse behavioral outcomes. Here we describe behavioral phenotypes resulting from perinatal nicotine exposure in a mouse model, and discuss our findings in the context of findings from previously published studies using preclinical models of developmental nicotine exposure.

**Methodology/Principal findings:**

Female C57Bl/6 mice received drinking water containing nicotine (100μg/ml) + saccharin (2%) starting 3 weeks prior to breeding and continuing throughout pregnancy, and until 3 weeks postpartum. Over the same period, female mice in two control groups received drinking water containing saccharin (2%) or plain drinking water. Offspring from each group were weaned at 3-weeks of age and subjected to behavioral analyses at 3 months of age. We examined spontaneous locomotor activity, anxiety-like behavior, spatial working memory, object based attention, recognition memory and impulsive-like behavior. We found significant deficits in attention and working memory only in male mice, and no significant changes in the other behavioral phenotypes in male or female mice. Exposure to saccharin alone did not produce significant changes in either sex.

**Conclusion/Significance:**

The perinatal nicotine exposure produced significant deficits in attention and working memory in a sex-dependent manner in that the male but not female offspring displayed these behaviors. These behavioral phenotypes are associated with attention deficit hyperactivity disorder (ADHD) and have been reported in other studies that used pre- or perinatal nicotine exposure. Therefore, we suggest that preclinical models of developmental nicotine exposure could be useful tools for modeling ADHD and related disorders.

## Introduction

It is estimated that approximately 37 million American adults and 3 million American middle school and high school students smoke cigarettes [1]. In addition, the use of electronic cigarettes (vaporized nicotine) is increasing, especially among young adults of reproductive age, due to false perceptions of increased safety. Between 2013 and 2014, in just one year, the use of e-cigarettes tripled among high school students [2]. Preclinical studies [3–9] and clinical studies [10–14] show that prenatal nicotine exposure or cigarette smoking by pregnant women is associated with an increased risk of cognitive disabilities in their children. Moreover, the adverse effects of nicotine exposure may not be limited to the nicotine exposed individuals alone but may be evident in up to two subsequent generations descending from the prenatally nicotine exposed individuals [8]. Therefore, the population at risk for the effects of prenatal nicotine exposure may be much larger than previously recognized. Thus, understanding the adverse effects of prenatal nicotine exposure, whether via conventional cigarettes, e-cigarettes or smokeless tobacco is a highly significant public health issue.

Preclinical models of developmental nicotine exposure have advanced our knowledge of the adverse effects of nicotine on the developing brain. However, variability in preclinical experimental design has led to inconsistent findings. For example, the nicotine formulation, route of nicotine administration, timing of nicotine exposure with respect to the stage of brain development, as well as the types of behavioral tests performed vary significantly among the different studies, even when the same species of experimental animals are used.

In fact, nicotine exposure can have different effects on the developing brain at different stages of brain development, and each developmental stage may be uniquely vulnerable. Our earlier studies [8, 9, 15] used a mouse model of prenatal nicotine exposure, in which the nicotine exposure began prior to conception and lasted until the day of birth. The prenatal period in mice corresponds approximately to the first two trimesters of human pregnancy with respect to brain development [16–19]. In the present study we extended the nicotine exposure period to 3 weeks postpartum, so the exposure occurred over a period corresponding approximately to the entire human gestation period [16–19].

Our data show that perinatal nicotine exposure produces some but not all of the cognitive phenotypes reported in our previous studies using prenatal nicotine exposure. When the present findings are examined in the context of findings from the studies in the literature, attention and working memory deficits emerge as the most consistent behavioral deficits associated with preclinical models of developmental nicotine exposure.

## Materials and methods

### Animals

C57BL/6 mice were purchased from Charles River Laboratories (Kingston, NY) and housed in the Florida State University laboratory animal care facility. The facility is a temperature and humidity controlled environment maintained on a 12-hr light-dark cycle (lights off at 7 AM and on at 7 PM) with food and water available *ad libitum*. Breeding age (8–12 week-old) female mice were randomly assigned to one of three experimental groups based on the type of drinking water supplied: nicotine + saccharin (N + S), saccharin only (S) or plain drinking water (W). The mice in the N + S group were provided with drinking water containing100 μg/ml nicotine ((-)-Nicotine, Sigma Chemical Company, St. Louis, MO; Cat# N3876) and 2% saccharin (Alfa Aesar, Heysham, England; Cat# A15530). Saccharin is used as a sweetener to mask the bitter taste of nicotine in the drinking water [8, 9, 15]. The mice in the S group received drinking water containing 2% saccharin, and the mice in the W group received drinking water without any additive. Following 3 weeks of such exposures, the female mice in each experimental group were bred with drug naïve male mice. The day of successful mating (verified by the detection of a vaginal plug) was designated embryonic day 0 (E0). Throughout pregnancy each female mouse was single housed. The three types of drinking water exposures continued until postnatal day (P) 21 when the offspring were weaned. To clarify, during the postnatal period (P0 to P21), the offspring were exposed to saccharin and nicotine via mothers’ breast milk. Some offspring may have consumed the drinking water directly from the water bottle, especially in the third postnatal week when they could have had direct access to the water bottles. The litter size was standardized to contain 6–8 offspring on the day of birth.

All of the experimental procedures were in full compliance with institutional guidelines at the Florida State University and the NIH *Guide for the Care and Use of Laboratory Animals*.

### Behavioral analyses

The behavioral analyses began when the mice reached postnatal day (P) 90. The offspring of the same sex from each of the three perinatal exposure groups were housed 2–4 per cage and were handled by the experimenter for 5 min/day for 3 days prior to the beginning of the behavioral analyses. Mice were habituated to the testing room for 30 min before the analyses commenced. The handling, habituation and behavioral testing occurred during the lights-off period, under dim lighting.

### Spontaneous locomotor activity

**Fig 1:**
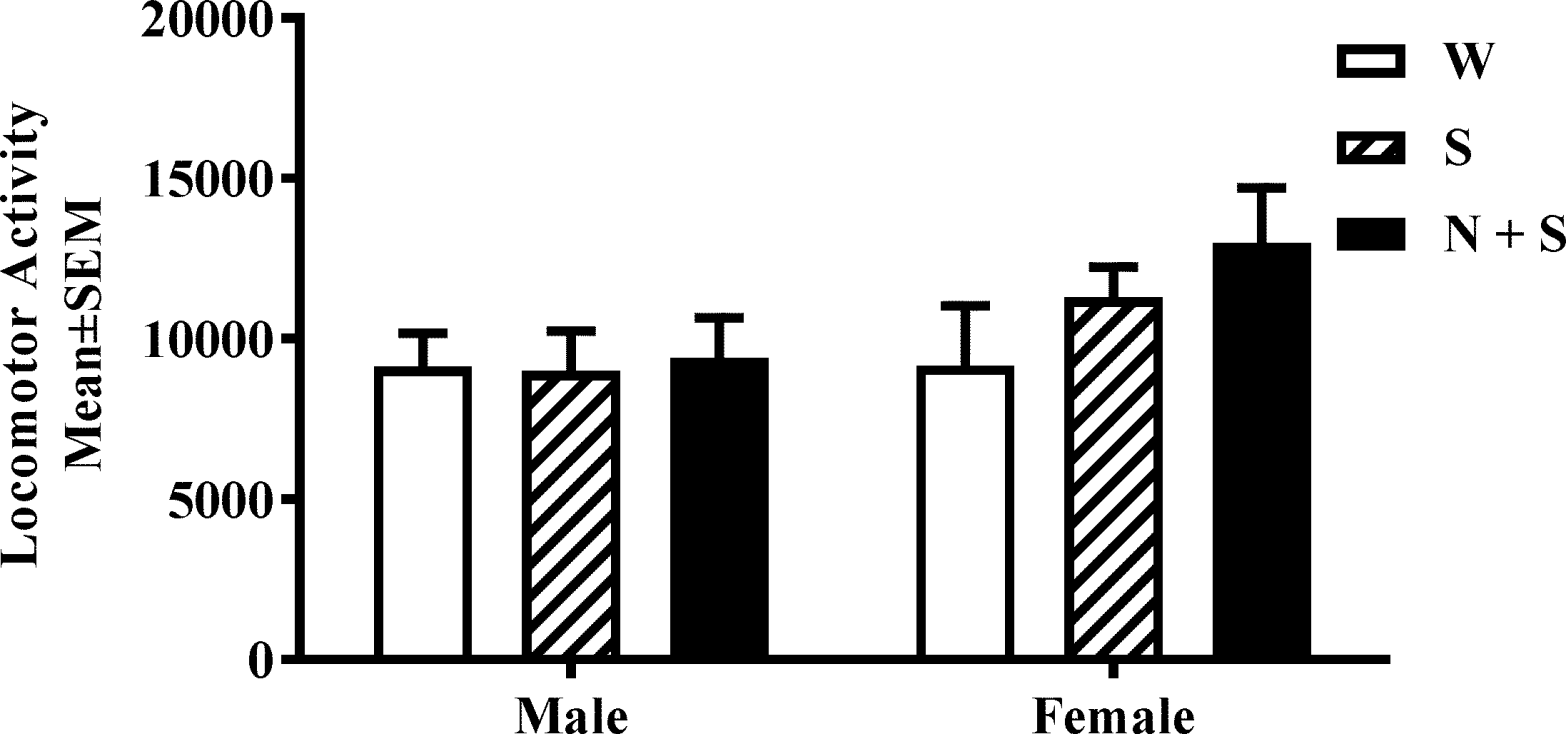
Perinatal nicotine exposure and spontaneous locomotor activity. Cumulative spontaneous locomotor activity was analyzed during the lights-off period (19:00–07:00 h) in male and female mice from the nicotine+saccharin (N + S), saccharin alone (S) and plain drinking water (W) groups. There was no significant difference in this measurement among the three experimental groups. [Mean ± SEM: Male; W = 9148 ± 1722; S = 9009 ± 1862; N + S = 9410 ± 1862; Female: W = 9172 ± 2048; S = 11284 ± 1783; N + S = 12394 ± 1983].

On the day of analysis, the mice were removed from their home cages, and placed individually in testing cages equipped with Photobeam motion sensors (Photobeam Activity System; San Diego Instruments). Each instance of consecutive break in adjacent photobeams (positioned 5.4 cm apart) was scored as an ambulatory event or activity. We analyzed activity during a 12 hr period from 19:00 hr to 7:00 hr (Fig 1), during the dark phase of the light-dark cycle.

### Spatial working memory

**Fig 2:**
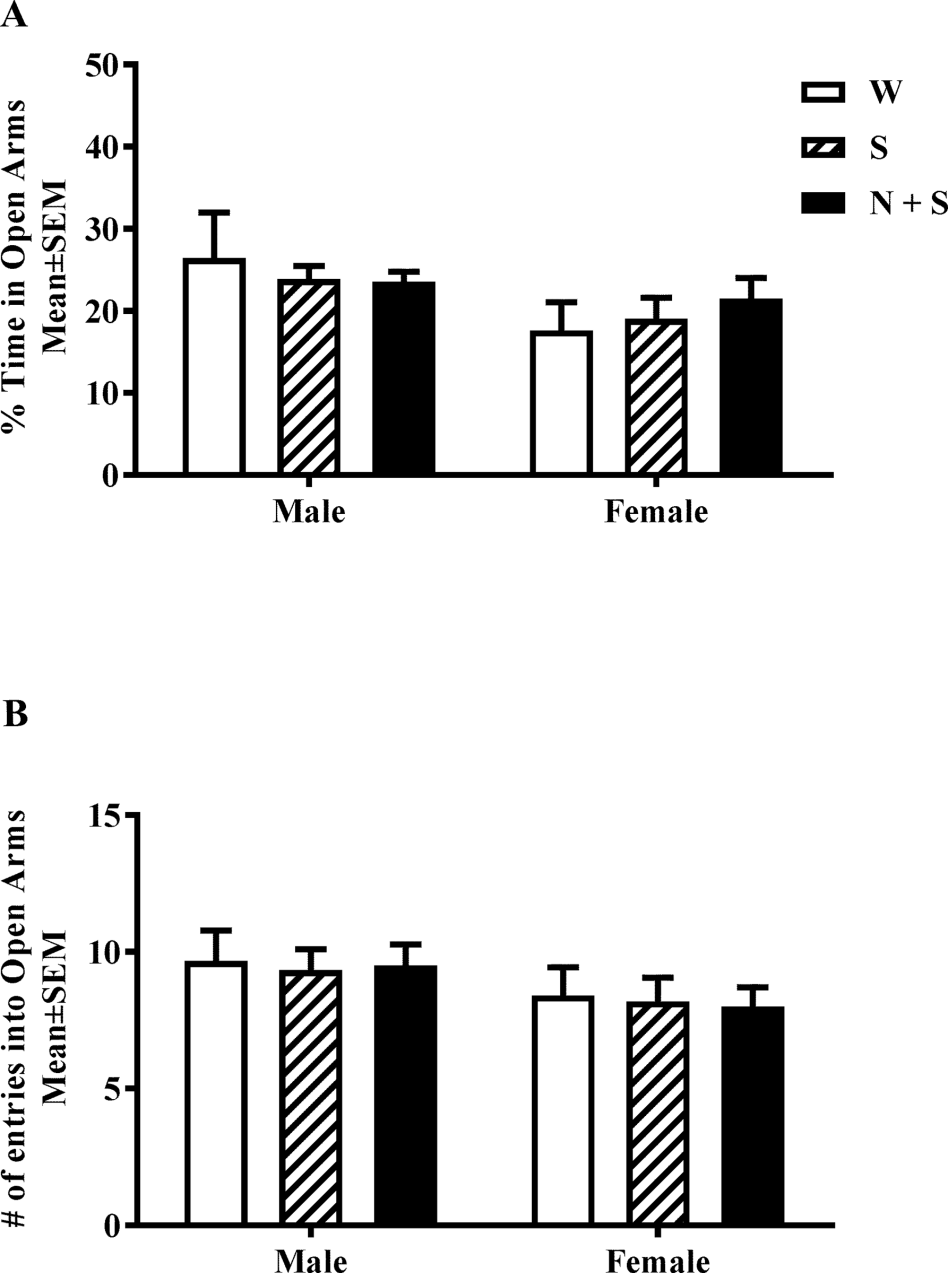
Perinatal nicotine exposure and anxiety-like phenotype in elevated plus maze (EPM). The percentage of time spent in the open versus closed arms (A) and the number of entries into open arms (B) of the EPM were analyzed. Neither measure showed significant difference in male or female mice from the nicotine+saccharin (N + S), saccharin alone (S) and plain drinking water (W) groups. [Mean ± SEM % time spent in open arms: Male: W = 26.44 ± 4.34; S = 23.89 ± 2.88; N + S = 23.56 ± 2.56; Female: W = 17.65 ± 3.88; S = 19.05 ± 23.84; N + S = 21.53 ± 3.64. Mean ± SEM number of entries into open arms: Male: W = 9.67 ± 1.21; S = 9.33 ± 1.17; N + S = 9.5 ± 1.16; Female: W = 8.40 ± 1.32; S = 8.20 ± 1.33; N + S = 8.00 ± 1.20].

Spatial working memory was assayed using a custom-built clear Plexiglas Y-maze consisting of three arms (each arm was 35 cm long x 6 cm wide x 10 cm high) of equal dimensions arranged in the shape of the letter “Y”. Unique visual cues were placed on the exterior of the walls of each arm as well as on the walls of the testing room to facilitate recognition of each arm as unique by the mouse. The mouse was placed at the center of the Y-maze, allowing free access to all 3 arms, and its behavior was recorded over a 10-min period using an overhead video camera. An investigator blinded to the identity of the mouse analyzed the video recordings to calculate the number of entries into each arm and the sequence of arm entries (for this purpose the arms were labeled A, B and C). An arm entry is considered to have occurred when all four limbs of the mouse enter an arm. A “spontaneous alternation” is defined as a set of three consecutive arm choices without a repeated entry (e.g. ABC, BCA, CAB) (Fig 2). A spontaneous alternation score was calculated using the formula: number of alternations / (number of entries—2) X 100.

### Elevated plus maze (EPM)

Anxiety-like behavior was analyzed using a standard mouse elevated plus maze (Med Associates, Inc., St. Albans, VT). The maze consisted of two open (50 cm × 10 cm) and two closed arms (50 cm × 10 cm with 40 cm walls) opposing each other, arranged in the shape of a plus sign (**+)**. The behavioral task was initiated by placing the mouse at the center of the maze facing one of the open arms with free access to the entire maze. Behavior was monitored via an overhead camera for a 5 min period. The time spent in the open arms and the closed arms, and the number of entries into open arms of maze were calculated from the video recordings.

### Object based attention (OBA)

The OBA test has been used as a test of attention in mouse models [4, 15, 20]. A rectangular Plexiglas box consisting of an exploration chamber (40 X 40 X 25 cm) and a testing chamber (40 X 20 X 25 cm) separated by a sliding door was used. The analysis began with a handling phase, in which the experimenter handled the mice 5 min/day for 3 days. On day 4, during the habituation phase, the mouse was placed in the empty exploration chamber for 5 min and then in the empty test chamber for another 5 min. The transit from the exploration chamber to the test chamber was permitted by lifting the sliding door. If a mouse did not voluntarily enter the test chamber, it was gently guided to do so. On day 5, during the acquisition session, the mouse was placed for 5 min in the exploration chamber containing five wooden objects of identical size but different shapes (rectangle, triangle, circle, oval and octagon). Next, the mouse was placed in the test chamber containing two wooden objects of the same size and shape (circle) for 5 min. The exploratory activity (defined as interactions with the object(s) from a distance of not greater than 1.5 cm) was recorded in each chamber. On day 6, the mouse was placed in the empty exploration chamber first and then in the empty test chamber, for 3 min each to permit habituation to the chambers. Next the mouse was returned to the exploration chamber, which now contained the same five objects that the mouse had explored on day 5. The exploratory activity was recorded for 3 min. Next the sliding door was opened and the mouse entered the test chamber, which now contained two objects. One object was of the same shape and size as one of the five objects the mouse had explored 3 min earlier in the exploration chamber (familiar object), and placed in the test chamber in a position analogous to its position of the object of the same shape in the exploration chamber. The second object was a star shaped “novel” object which it has not previously encountered before. Although the mouse had not been exposed to either of these two objects, it had been exposed to an object of the same size and shape as the “familiar” object, but had never been exposed to the “novel” object or any other star shaped object previously. The mouse explored the two objects for 3 min, and exploratory activity was recorded.

Preliminary analyses showed that the mice explored all four corners of the exploration and test chambers equally. Therefore, on days 5 and 6, the two objects were placed in the test chamber equidistant from the middle of the sliding door. The position of the objects in the exploration and test chambers was fixed throughout the entire study. The familiar object was always a circle and the novel object a star throughout the entire study.

The day 5 data were used to evaluate inclusion/exclusion criteria. Since all mice met the inclusion criterion of exploring all objects in the exploration and test chambers on day 5, we did not have to exclude any mouse from the study. Initial analysis revealed that the time spent exploring the 5 objects by the mice in the three treatment groups was not significantly different. During the test session, an exclusion criterion of <10% (i.e. < 18 s with both objects) was used based on our own previous studies and data from other studies [3, 4, 15]. All mice met this criterion, and therefore no mouse was excluded.

A recognition index was calculated using the formula: TN / (TF + TN) X 100, where TF and TN are time spent during the test session exploring the familiar and the novel objects, respectively, by an investigator blinded to the identity of the mouse.

### Novel object recognition (NOR)

We used the NOR test to evaluate recognition memory [21, 22]. The analysis began with the experimenter handling the mice 5 min/day for 3 days (days 1–3). On days 4 and 5 the mouse was placed in the test chamber (32 × 28 × 30 cm) for 20 min for habituation to the chambers. On day 6 the mouse was placed for 5 min in the test chamber, which now contained two identical objects selected at random from a collection of 2 sets of identical objects: Two unopened and unmarked cans of food (3.14 X 3.6 X 11 cm) or two Lego objects (6.4 X 6.4 X 11 CM). The two sets are needed so that in the next stage of the test, a novel object can be drawn from a set to which the mouse was not previously exposed (see below). Total time spent exploring the objects as well as the time spent exploring the object placed on left versus right side was calculated. These data were used *post hoc* to evaluate object or side bias. A counter-balanced design was used to address potential bias.

Next, the mouse was returned to its home cage. Following 10 min in the home cage, the mouse was returned to the test chamber, which now contained one of the previously explored objects and a novel object (chosen from the set not used in the previous step). The mouse was allowed to explore the objects for 5 min. The total time spent exploring both the objects as well as time spent exploring each object were calculated.

During each stage of the test (except during the 10 min stay in the home cage), the behavior of the mouse was recorded using an overhead video camera. An investigator blinded to the identity of the mouse analyzed the video recordings. Based on an exclusion criterion of less than 30 sec exploration of objects (i.e. <10% of the allotted time) no mouse was eliminated from the study.

The novel object recognition index was calculated using the formula: time spent with the novel object on day 6 / the time spent with both the objects on day 6 (novel object + familiar object) X 100.

### Cliff avoidance reflex (CAR)

Impulsive-like behavior was assayed using an apparatus consisting of a custom-built round Plexiglas platform (20 cm in diameter) supported on a plastic rod (50 cm in height) similar to a barstool [23]. The mice were placed individually on the platform and their behavior was recorded via an overhead video camera for a period of 60 minutes. When a mouse fell off the platform, it was gently picked up and returned to the platform. An investigator blinded to the identity of the mouse analyzed the video recording to calculate the average length of time to first fall (latency to first fall) during the 60 min interval.

### Number of mice used and the sequence of behavioral tests

Male and female mice from up to 8 litters from each of the three treatment groups (N+S, S and W) were used in each of the behavioral paradigms. A total of 6 behavioral tests were conducted in this study with approximately one-week interval between the tests. Using a mouse in every one of the 6 tests sequentially would have meant that the mouse would have been approximately 2 months older by the end of test #6 compared to their age during test #1. Moreover, we were concerned that the experience of undergoing more than 3 behavioral tests consecutively could influence performance in the subsequent behavioral tests. To avoid the potential influence of these variables, one male and one female mouse from each litter were assigned to one of two groups. Each group of mice was tested in only one battery of behavioral tests. Thus, the first group was examined sequentially in spontaneous locomotor activity, EPM and NOR test, and the second sequentially in Y-maze, OBA test, and CAR test. The NOR test was conducted in male mice only.

### Statistical analysis

We confirmed normal distribution of data from all our experiments. Therefore, differences between experimental groups were analyzed for statistical significance using parametric statistical tests. Two-way ANOVA was used to test the main effects of perinatal treatment and sex, and the interaction between these two factors. When a significant difference (*p*<0.05) was found by ANOVA, Bonferroni multiple comparison *post hoc* test was used. A one-way ANOVA was used for analysis of the NOR data as comparisons were made among the three experimental groups for male mice only. An unpaired Student’s *t*-test was used whenever differences between only two groups were evaluated. Data on bodyweight from P0 to P21 were analyzed using Repeated Measures ANOVA. In all cases, a *p* value of < 0.05 was considered to be statistically significant. Prism 6 Software (GraphPad Software Inc., San Diego, CA) was used for the statistical analyses.

## Results

### Body weight, water consumption, length of pregnancy, litter size, and offspring metrics

There was no significant difference in body weight among the three treatment groups over the period P0 to P21 [F(2,60) = 0.002, *p* = 0.99]. However in the case of body weight at P90, there was a significant main effect of sex [F(1,42) = 770, *p*<0.0001], and no significant main effect of treatment [F(2,42) = 0.51, *p* = 0.60] or interaction between sex and treatment [F(2,42) = 0.05, *p* = 0.95]. Bonferroni multiple comparison *post hoc* test showed that the P90 male mice had significantly greater body weight compared to females, regardless of the perinatal treatment [S1 and S2 Tables].

The average length of pregnancy (19–20 days), litter size at birth (6–9), and sex ratio at birth (1:1) were comparable among the three treatment groups. Drinking water intake by pregnant dams in the three treatment groups throughout the experimental period (3 weeks prior to mating and during pregnancy) was also comparable [Mean ± SEM (ml/day): W: 9.86 ± 0.66; S: 10.59 ± 0.97; N + S: 9.47 ± 0.89]. The following developmental milestones were achieved on average at the same time by all offspring in the three treatment groups: Ears detached on P4, fur appeared on P6-7 and eyes opened on days P14-15. The developmental milestones are comparable to those in our previous studies of prenatal nicotine exposure, in which the offspring were cross-fostered to drug naïve dams on the day of birth [8, 15].

### Spontaneous locomotor activity

There were no significant main effects of treatment [F(2,44) = 1.09, *p* = 0.35] or sex [F(1,44) = 3.19, *p* = 0.08], or interaction between sex and treatment [F(2,44) = 0.85, *p* = 0.43 [Fig 1; S1 Table].

### Elevated plus maze (EPM)

There were no significant main effects of treatment [F(2,27) = 0.06, *p* = 0.95], sex [F(1,27) = 3.92, *p* = 0.06], or interaction between sex and treatment [F(2,27) = 0.56, *p* = 0.58] on time spent in open arms [Fig 2A, S1 Table]. Similarly, there were no significant main effects of treatment [F(2,27) = 0.06, *p* = 0.94], sex [F(1,27) = 3.16, *p* = 0.09], or interaction between sex and treatment [F(2,27) = 0.02, *p* = 0.98] on the number of entries into the open arms [Fig 2B, S1 Table].

### Spatial working memory

**Fig 3:**
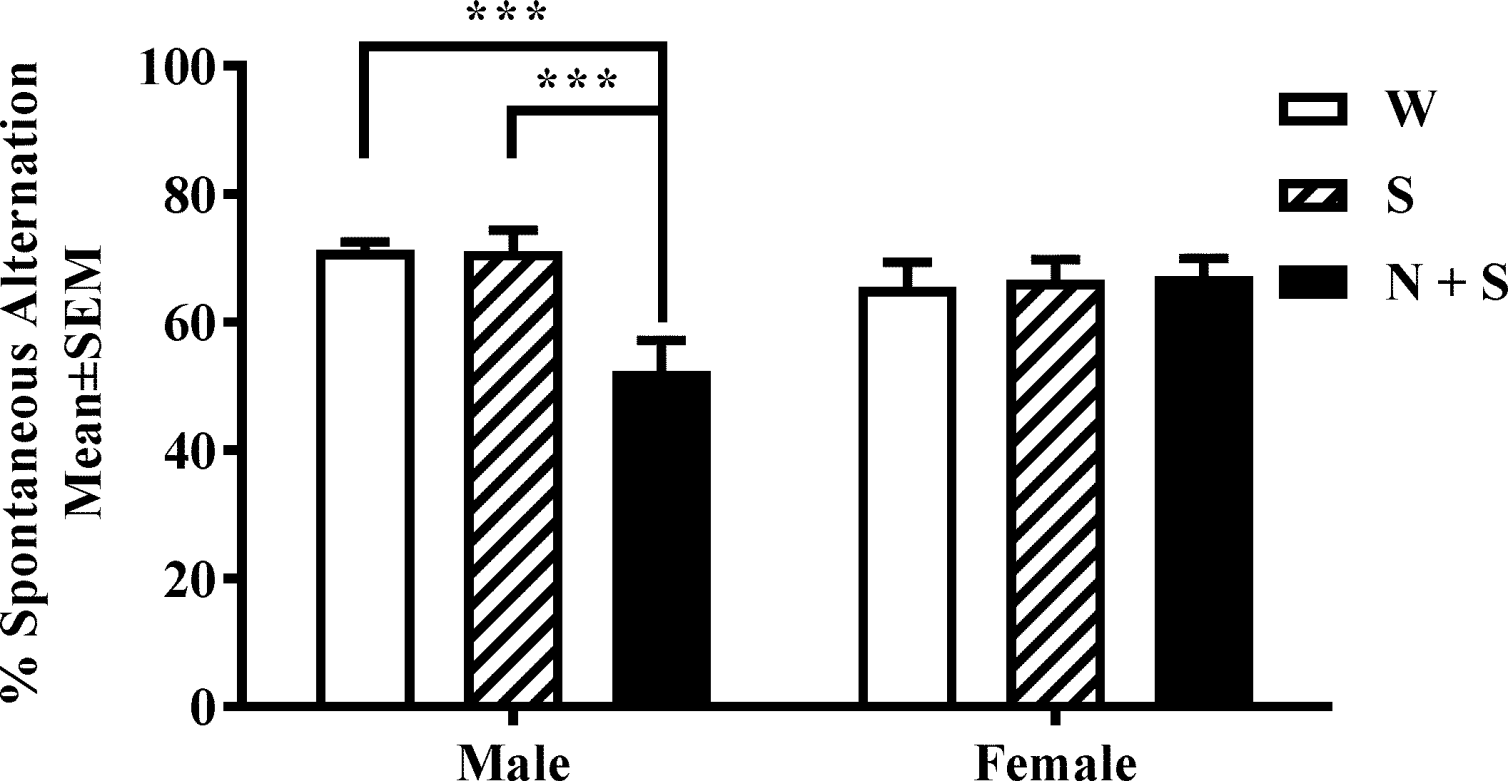
Perinatal nicotine exposure produces a significant decrease in spontaneous alternation in the Y-maze in male but not female offspring. Spontaneous alternation in the Y-maze was analyzed in male and female mice from the nicotine+saccharin (N + S), saccharin alone (S) and plain drinking water (W) groups. There was a significant decreases in this measurement in male mice from the N+S group. ***p<0.001. [Mean ± SEM: Male: W: 71.31 ± 1.20; S: 71.11 ± 4.33; N + S: 52.37 ± 4.63; Female: W: 65.57 ± 4.43; S: 66.63 ± 4.42; N + S: 67.19 ± 3.56].

There was a significant main effect of treatment [F(2,32) = 5.01, *p* = 0.01] and interaction between sex and treatment [F(2,32) = 6.37, *p* = 0.0047], and no significant main effect of sex [F(1,32) = 0.34, *p* = 0.56] on spontaneous alternation in the Y-maze. Bonferroni multiple comparison test showed significant decreases in spontaneous alternation in male mice in the N+S group compared to the male mice in the W or S groups [Fig 3]. None of the other pair-wise comparisons were significant [S1 and S2 Tables].

### Object based attention (OBA)

**Fig 4:**
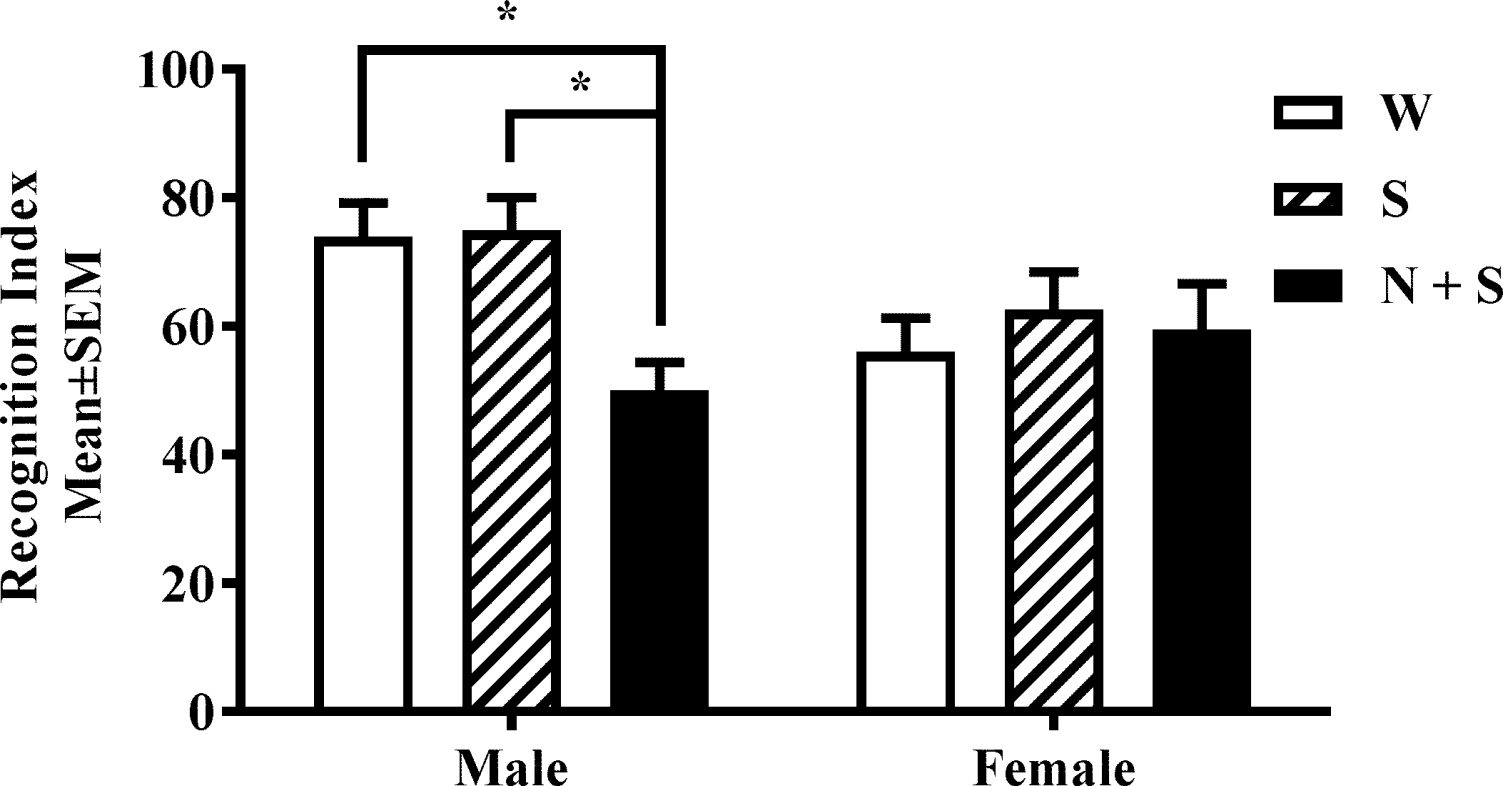
Perinatal nicotine exposure produces attention deficits in male offspring in the object based attention (OBA) test. Recognition index was analyzed in male and female mice from the nicotine+saccharin (N + S), saccharin alone (S) and plain drinking water (W) groups. There was a significant decrease in this measurement in male mice from the N+S group. *p<0.05. [Mean ± SEM: Male: W: 73.95 ±4.65; S: 74.93 ± 4.85; N + S: 50.04 ± 4.65; Female: W: 56.01 ± 4.60; S: 62.61 ± 4.80; N + S: 59.46 ± 7.85].

The recognition index was >50% in male and female mice in the W and S (control) groups [Fig 4] suggesting a greater than chance performance level in the control groups. However, to establish that the control groups “recognized” the novel object over the familiar object on Day 6 (test day), we compared time spent exploring the familiar *versus* the novel object by male and female mice in the W and S groups. In both the control groups, male and female mice spent significantly longer time exploring the novel object compared to the familiar object (Male W: t = 6.41, *p* = 0.001; Female W: t = 2.35, *p* = 0.04; Male S: t = 6.93; *p* = 0.001; Female S: t = 3.06, *p* = 0.01) indicating that the mice in the control groups successfully performed the test. Next, we analyzed the data for all treatment groups. There was a significant main effect of treatment [F(2,30) = 3.41, *p* = 0.04] and interaction between sex and treatment [F(2,30) = 3.39, *p* = 0.04], and no significant main effect of sex [F(1,30) = 2.35, *p* = 0.14] in the OBA test. Bonferroni multiple comparisons test showed significant deficits in OBA in male mice in the N+S group compared to their counterparts in the W and S groups. None of the other pair-wise comparisons showed significant effects [Fig 4, S1 and S2 Tables].

### Novel object recognition (NOR)

**Fig 5:**
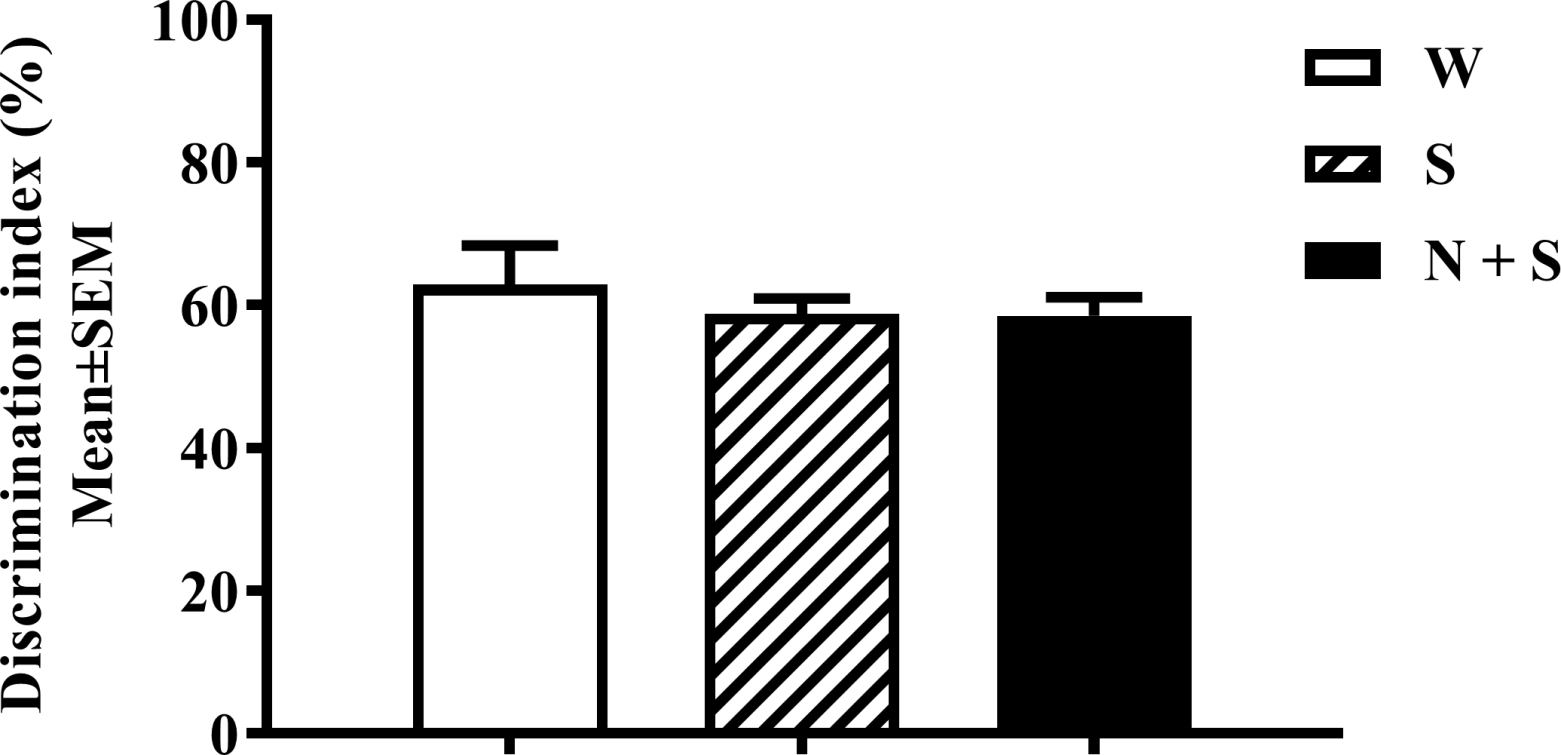
Perinatal nicotine exposure and Novel-object recognition test (NOR). Data combined from the nicotine+saccharin (N + S), saccharin alone (S) and plain drinking water (W) groups are shown There was no significant difference in the time spent exploring novel versus familiar object (discrimination index) in male and female mice from the N + S, S and W groups. [Mean ± SEM: Male: W = 69.92 ± 5.26; S = 58.8 ± 1.11; N + S = 58.57 ± 2.11.

Since only male mice in the N+S group showed significant deficits in the OBA test [Fig 4, S1 and S2 Tables], we used only male mice in the NOR test. Initially, we examined whether the mice showed left-right bias. A Student’s *t* -test showed no significant bias toward a side [Mean±SEM Exploration (sec): Left: 21.3 ± 1.33; Right: 22.39 ± 1.15; t = 0.62, df = 46, *p* = 0.54], or toward an object [Mean±SEM Exploration (sec): Un-opened can: 43.15 ± 4.15; Lego: 44.24 ± 2.54; t = 0.22, df = 22, *p* = 0.82]. Next, we compared time spent exploring the familiar versus novel object by male mice in the W and S control groups. In both the control groups, the mice spent significantly longer period of time exploring the novel object compared to the familiar object (W: t = 3.33, *p* = 0.01; S: t = 5.77, *p* = 0.01). Finally, a one-way ANOVA showed no significant difference among the three treatment groups in the novel object recognition index [F(2,21) = 0.43, *p* = 0.65; Fig 5].

### Cliff avoidance reflex (CAR)

**Fig 6:**
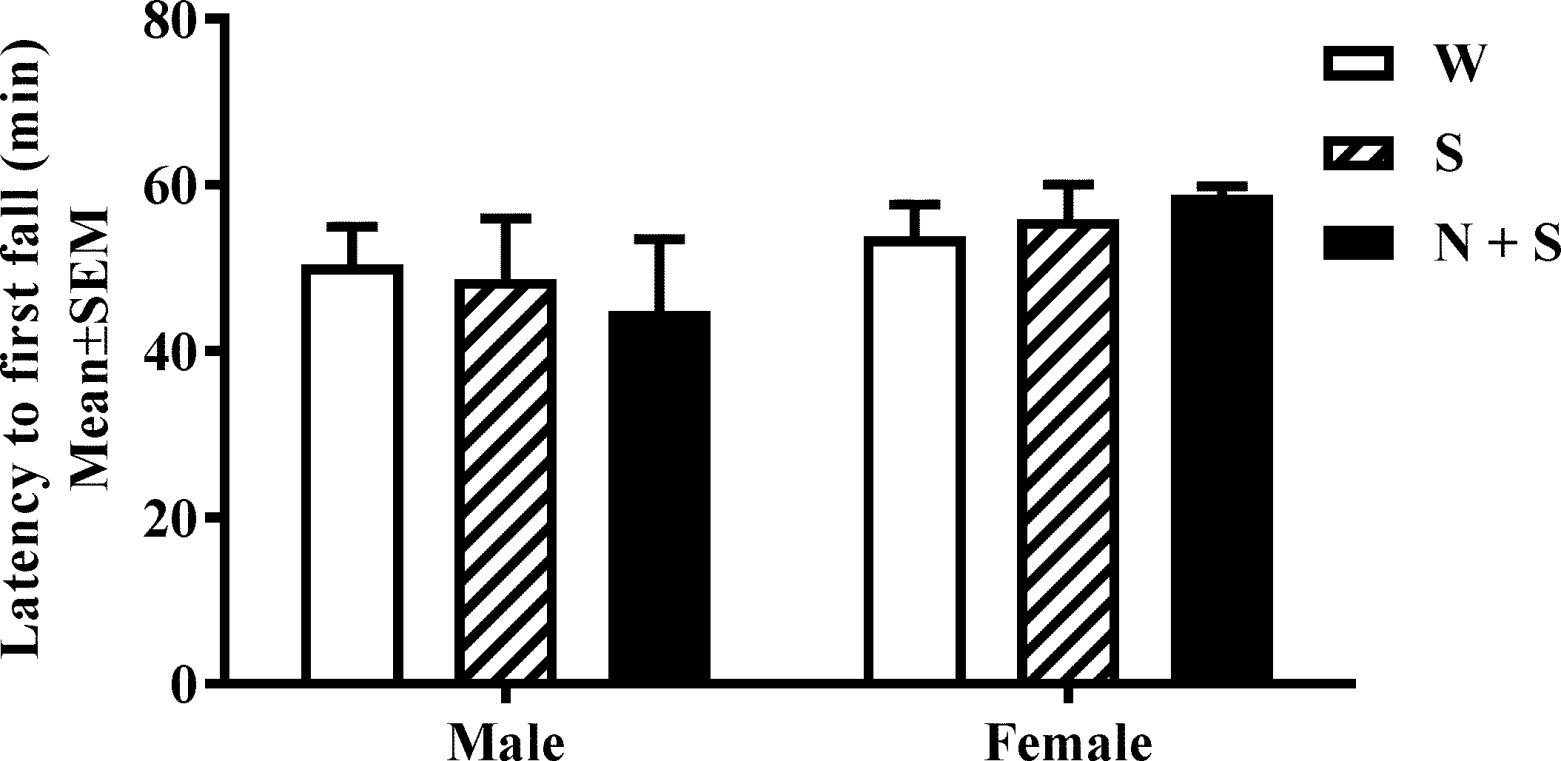
Perinatal nicotine exposure and cliff avoidance reflex (CAR). The latency to first fall over the 60 min interval were analyzed. There was no significant difference in the latency to first fall [Mean ± SEM (min): Male: W = 50.41 ± 3.83; S = 48.67 ± 5.56; N + S = 44.84 ± 7.78; Female: W = 53.83 ± 2.91; S = 55.92 ± 3.01; N + S = 58.83 ± 2.08] in male or female mice from the nicotine+saccharin (N + S), saccharin alone (S) and plain drinking water (W) groups.

A two-way ANOVA of the latency to first fall data did not show significant main effects of treatment [F(2,30) = 0.003, *p* = 0.99], main effect of sex [F(1,30) = 3.35, *p* = 0.08], or interaction between sex and treatment in the CAR assay [F(2,30) = 0.47, *p* = 0.63] [Fig 6, S1 Table].

## Discussion

Our data show that perinatal nicotine exposure beginning 3 weeks prior to conception, continuing through gestation, and ending at weaning produces significant deficits in spatial working memory (Y maze) and attention (OBA) at P90 in male offspring but not in female offspring. The perinatal nicotine exposure did not produce significant changes in spontaneous locomotor activity, anxiety-like behavior (EPM), impulsive-like behavior (CAR), or recognition memory using novel object recognition (NOR) at P90. The nicotine exposure did not produce significant effects on litter size, offspring birth weight, weight gain during development or mature body weight at P90. Exposure to saccharin alone did not influence any of the parameters.

The deficits in attention and working memory in male mice emerge as the only significant behavioral deficits produced by the perinatal nicotine exposure in the present study. Interestingly, working memory and attention deficits were also fund in our prenatal nicotine exposure mouse model [15], and are comorbid conditions in ADHD [24–28]. Therefore, developmental nicotine exposure mouse models appear to have significant face validity as ADHD experimental models.

We used the OBA test to evaluate attention in the present study and in our previous study of prenatal nicotine exposure [15]. The OBA test has been used to assay attention in other mouse models of developmental nicotine exposure [3, 20]. Although the OBA test incorporates design features intended to exclude recognition memory as a confounding variable [15, 20], there may be a concern that performance in the OBA test by the N+S group of mice may be influenced by potential direct effects the nicotine exposure on recognition memory. To address this potential concern, we measured recognition memory using the NOR test. Consistent with findings from other studies [4], we found that male mice in the N+S group did not show significant changes in recognition memory compared to the control groups. Therefore, the attention deficits in the OBA test observed in the male mice (female mice did not show OBA deficits) are unlikely to be influenced by changes in recognition memory.

Another major finding in the present study is that the deficits in attention and working memory are sex-specific. Sex differences in nicotine’s effects on the brain and behavior have been described previously [29–32]. Developmental nicotine exposure produces sex-dependent changes in hyperactivity, nicotine preference and pre-pulse inhibition [6, 33–36]. A review of the literature shows that sex differences in hypothalamic-pituitary axis signaling, estrogen receptor signaling, neurotransmitter receptor signaling, especially dopamine receptor expression are among the candidate mechanisms proposed for sex differences in the effects of developmental nicotine exposure [30, 31, 37, 38]. Another potential factor contributing to sex-dependent effects of developmental nicotine exposure may be nicotine-induced epigenetic modification of the DNA and histones. Since the nicotine exposure in the present study began prior to conception, it is possible that nicotine produced epigenetic modification of the DNA and histones in the placenta and somatic cells of the offspring during pre- and postnatal development. Smoking during pregnancy alters DNA methylation in the placenta [39, 40] and somatic cells of the offspring [41, 42]. In addition, nicotine can have a direct impact on DNA methylation in somatic cells [43, 44] of the exposed individual. Equally interestingly, developmental exposure to cotinine, a metabolite of nicotine can also produce epigenetic modification of the DNA [45]. Epigenetic modifications can promote or repress gene expression, and some of the epigenetic changes can influence sex-specific gene expression. Since there are imprints/parent-of-origin effects on transcription at over 1300 loci [46, 47] and there are ~350 autosomal genes with sex-specific parent-of origin effects in the mouse brain [46, 47], it is conceivable that the sex-specific effects of developmental nicotine exposure have their origins in sex-specific gene transcription during development. The role of genetic sex, and organizational *versus* activational influences, imprinted genes and mitochondrial DNA [46, 47] in mediating sex-specific effects of nicotine exposure remain to be elucidated.

It has been suggested that the sex-dependent effects of developmental nicotine exposure on behavioral parameters may be secondary to nicotine’s sex-specific effects on physical development. In one study, prenatal nicotine exposure significantly reduced body weight of the female offspring during neonatal and pre-pubertal development, whereas the growth retardation in the male offspring occurred later in the adolescent period [48]. The smaller size of the female offspring in the early postnatal period could impact mother-infant interactions, and thereby influence behavioral development selectively in the female offspring. Furthermore, prenatal nicotine exposure increased anxiety-like behavior at P40 in male but not female offspring [48, 49]. However, in the present study we did not observe significant effects of nicotine on body weight gain in the offspring.

The lack of attention deficits in female mice in the present study is consistent with findings from human studies, where the diagnosis of ADHD in boys is nearly twice as frequent as that in girls, although girls and boys manifest the same symptoms [50].

In the present study, the effects of perinatal nicotine exposure on behavioral parameters are more “selective” or less “severe” than the effects of prenatal nicotine exposure we had reported previously [9, 15]. This conclusion seems counterintuitive because the perinatal nicotine exposure involves a longer exposure period and therefore may be expected to produce more “severe” behavioral outcomes compared to the prenatal exposure. Multiple factors could contribute to these seemingly counterintuitive findings.

Timing of the nicotine exposure *vis a vis* the stages of brain development is a major factor in determining the effects on the brain and behavior. For example, nicotine exposure occurring from the 12^th^ day of gestation until birth was reported to produce significant effects on cognitive and emotional behaviors in contrast to exposure occurring prior to this date, which was reported to be ineffective [3, 4]. However, in our pre- and perinatal exposure models, the nicotine exposure began at the same time, prior to conception, suggesting that the length of the exposure may be an equally significant parameter.

One possibility is that with longer nicotine exposures, the potential for adaptation within the neural systems is proportionately greater. For example, the nicotinic acetylcholine receptor signaling mechanisms may respond differently to early *versus* late onset as well as short *versus* long duration of exposures. Expression of nearly all of the nicotinic acetylcholine receptor subtypes in the brain begins early in the embryonic period and the receptors undergo significant cell-type and brain region specific remodeling throughout pre- and postnatal development [51–58]. It is conceivable that the timing of onset and duration of the nicotine exposure impact receptor signaling differently. In addition, if the nicotine exposure began at prenatal stages and continued into early postnatal stages of development, the prenatal effects may modify the later postnatal effects. One possibility relevant to the present data is that the postnatal exposure somehow “mitigates” the effects of the prenatal exposure such that the combined effects are less severe than the effects of prenatal or postnatal exposures alone.

Besides the “timing” of the nicotine exposure, cross fostering could have been a variable between our prenatal and perinatal nicotine exposure models. Cross fostering is utilized in preclinical models of developmental nicotine exposure to limit the nicotine exposure to prenatal period as well as to avoid potential adverse effects of impaired interactions between nicotine-exposed dams and their offspring upon the body and brain development of the offspring [6–9, 15, 49, 59]. Only one previous study [49] directly examined the impact of cross fostering in a rat model of prenatal nicotine exposure, and found that cross fostering had significant impact on open field exploratory activity and elevated plus maze but not on learning and memory. In our prenatal nicotine exposure paradigm [8, 9, 15], although we used cross fostering, mice in the nicotine-exposed group as well as the controls groups were cross fostered, in an attempt to control for the effects of cross fostering. However, our experimental design could not have addressed any differential between the impacts of cross fostering on the control versus the nicotine-exposed groups.

Cross fostering introduces sudden withdrawal from nicotine as a variable, as the supply of nicotine from the nursing mothers’ milk becomes abruptly curtailed upon cross fostering to a non-nicotine exposed dam. Nicotine withdrawal becomes a variable in virtually every preclinical model of prenatal nicotine exposure because even when the offspring are not cross fostered, access to nicotine is abruptly terminated at the time of weaning. One study used a gradual step-up and step-down method of nicotine exposure [60] to avoid the impact of abrupt nicotine exposure and withdrawal. However, the effects of abrupt *versus* gradual withdrawal on the developing brain or behavioral phenotypes were not compared in the study.

Another behavioral phenotype observed in our prenatal nicotine exposure model and not present in the present model is CAR, a measure of impulsivity [23]. We found that although the prenatally nicotine exposed male mice showed significant impairment in CAR [15], neither male nor female mice showed significant changes in the present study. Impulsive decision making behavior without regard to the “value” of the reward [44] and decreased pre-pulse inhibition [36] has been reported in rat models of developmental nicotine exposure.

Although the Discussion above focused primarily on the differences between the findings from our prenatal *versus* perinatal nicotine exposure paradigms, a review of the literature underscores the variability in behavioral outcomes from different mouse models of pre- or perinatal nicotine exposures [Tables 1 and 2]. Although the precise mechanisms that could contribute to the differences remain a topic of discussion, a variety of experimental variables emerge as potential contributors to the differences. These variables include the mouse strain used (C56Bl/6, SW, DAB), type of nicotine used (freebase, tartrate, hydrochloride), route of administration (via drinking water, subcutaneous infusion pump, intraperitoneal, intravenous self-administration), age at which the behavior was examined (20 to 180 days of age), and the behavioral methodologies used [Tables 1 and 2]. The following discussion may serve to place the differences between our pre- and perinatal nicotine exposure models within this broader context.

**Table 1:** Summary of the literature on the effects of developmental nicotine exposure on anxiety-like behavior in mice.

Mouse Strain	Nicotine Exposure	Assay	Finding	Citation
Swiss Webster	Nicotine freebase 0.5 mg/kg; s.c. daily from E0 to P0	Elevated plus maze in "weaned" mice. Males were used	Increased time and increased entries into open arms	[30]
				
C57BL/6J (SLC Inc., Shizuoka, Japan)	Nicotine freebase 200 μg/ml in drinking water containing 2% sucrose from various times during gestation until various times in the postnatal period	Light-dark box at P26-P38. Males and females were used	Decrease in time spent in lighted box in males exposed to nicotine from E0-P0 and E14-P0. No effect in females	[3]

		Elevated plus maze at P26-P38. Males and females were used	Significant reduction in time spent in open arms in males exposed to nicotine from E0-P7, E14-P7, E0-P0 and E14-P0. In females similar reduction was found when they were exposed from E0-P0. No effect on the number of open arms entries in entries males or females	
				
C57BL/6J	Inhalation of cigarette smoke 6 hr/day during gestation until weaning	Light-dark box at P90. Males and females were used	Longer duration in the lighted area	[31]
		Elevated zero maze at P90. Males and females were used	Increased time and increased entries into open arms only in males	
				
DBA/2J and C57BL/6J	Nicotine freebase in drinking water 200μg/ml starting 30 days before mating and continuing through gestation until weaning	Elevated plus maze on P24 and P75. Male and female mice were used	Increased entries into open arms at P75 in DBA/2J female mice and longer time in closed arms at P75 in C57Bl/6j male mice	[32]
				
CD1	Nicotine free base 4 mg/kg s.c. daily in 2 doses from E0 to P0	Elevated plus maze at P180. Females were used	No change in time spent in open versus closed arms	
		Elevated platform test at P180. Females were used	Increased time on platform (increased anxiety-like behavior)	[34]
		Suok test at P180. females were used	Decreased sensorimotor integration (greater number of missteps); increased anxiety	
				
C57BL/6J	E-cigarette vapor containing 2.4% nicotine 20 min/day from E15-19 and again from P2-16	Light-dark box at P98. Males were used	Longer duration and number of entries in the lighted area	[33]
		Elevated zero maze at P98. Males were used	No effect on time spent in open versus closed arms and increased number of head dips in open arms	

**Table 2:** Summary of the literature on the effects of developmental nicotine exposure on locomotor activity in mice.

Mouse Strain	Nicotine Exposure	Assay	Finding	Citation
Swiss Webster	Nicotine freebase 0.5 mg/kg; s.c. daily from E0 to P0	Locomotor Activity in open field at P31. Male mice were used	Hyperactivity	[61]
				
C57BL/6J	Inhalation of cigarette smoke 6 hr/day during gestation until weaning	Locomotor Activity in open field on P90. Male and female mice were used	Hyperactivity in the initial 5 min and Hypo-activity at later times	[62]
				
DBA/2J and C57BL/6J	Nicotine freebase in drinking water 200μg/ml starting 30 days before mating and continuing through gestation until weaning	Locomotor Activity in open field on P24 and P75. Male and female mice were used	Hyperactivity in DBA/2J at P24 and P75 and in C57BL/6J mice at P24. Hypo-activity in C57BL/6J females at P75	[63]
				
Swiss Webster	Nicotine hydrogen tartrate (200μg/ml) in drinking water containing 2% saccharin starting 2 weeks before mating and continuing through gestation until weaning	Locomotor Activity in open field at 3–10 weeks of age. Male mice were used	No effect	[68]
				
C57BL/6J	Nicotine hydrogen tartrate (200μg/ml) in drinking water containing 2% saccharin through gestation until weaning	Locomotor Activity in open field on P31-32. Male and female mice were used	Increased locomotor response to novel environment but no effect later	[66]
				
C57BL/6J	Nicotine freebase 200μg/ml in drinking water containing 2% saccharin starting 30 days before mating and continuing through gestation	Locomotor Activity in open field on P20, P40, and P60. Male and female mice were used	Females were hypoactive on P20. Males were hyperactive on P40 and P60	[6]
				
C57BL/6J	Nicotine hydrogen tartrate 0.05 mg/ml drinking water containing 0.066% saccharin starting 2 weeks prior to conception and continuing through gestation and until weaning	Locomotor Activity in open field from P60-P100. Male and female mice were used	Hyperactivity	[60]
				
C57BL/6J (NCI)	E-cigarette vapor containing 2.4% nicotine 20 min/day from E15-20 and again from P2-16	Locomotor Activity in open field at P98. Male mice were used	Hyperactivity	[64]
				
B6C3F1	Inhalation of cigarette smoke 4 h/d and 5 d/week from E4 until E18	Locomotor Activity in open field at P28 and P120. Males and females were used	Hyperactivity in both sexes at P28, and only in males at P120	[67]
				
C57BL/6	Nicotine freebase 200 μg/ml in drinking water containing 2% saccharin beginning 3 weeks before conception and continuing until P0	Spontaneous locomotor activity in non-home cage measured continuously over 20 hr in P42-P60 male and female mice.	Hyperactivity in males and females	[9]

We will use as examples two commonly evaluated behavioral phenotypes namely, anxiety-like behavior and locomotor activity to compare the findings from the different experimental paradigms. Anxiety-like behavior has been analyzed using open field exploration, EPM, elevated zero maze or light-dark box. The findings from some of these studies [Table 1] show increased time spent in the open arms or lighted areas, suggesting a more exploratory and less anxious phenotype [61–64], and the others show the opposite [4, 63, 65]. Locomotor activity was analyzed using continuous measurements of motor activity in a novel environment (open-field or a rodent cage) for 30 min to 2 hr, or continuous measurements of activity over 20–24 hr in the home cage or a novel cage [Table 2]. In other cases, number of entries into open or closed arms of the EPM or the arms of a Y maze was also used as a measure of locomotor activity [Table 1]. Overall, some studies reported increased locomotor activity [6, 60–63, 66, 67], whereas others reported decreased activity [62, 63] or no significant change [68]. The most significant biological variables in the studies compiled in Tables 1 and 2 may be the timing of the nicotine exposure with respect to the stage of brain development [16–19], and the mouse strain used.

In conclusion, deficits in working memory and attention in male mice remain the consistent findings in our present study of perinatal nicotine exposure and our previous study of prenatal nicotine exposure. Although these two cognitive parameters are not among the most commonly evaluated parameters in preclinical models of developmental nicotine exposure in the literature, the reports that did evaluate these parameters have found deficits in both phenotypes. Since working memory and attention deficits are found in ADHD, we suggest that preclinical rodent models of developmental nicotine exposure can serve as useful models of behaviors associated with ADHD. Finally, a review of the behavioral findings in the literature from preclinical models of developmental nicotine exposure shows that experimental design variations can contribute significantly to the direction and magnitude of the behavioral outcomes.

## Supporting information

S1 Table Two-way analysis of variance data for behavioral phenotypes. (DOCX)

S2 Table Bonferroni post hoc multiple comparison data for behavioral phenotypes. (DOCX)

## Abbreviations

ADHD: Attention deficit hyperactivity disorder
OBA: Object Based Attention
CAR: Cliff Avoidance Reflex
EPM: Elevated Plus Maze
N+S: Nicotine + saccharin
S: Saccharin alone
W: Plain drinking water
