# Supplementary material for: Attention and working memory deficits in a perinatal nicotine exposure mouse model
Source: PLoS One. 2018 May 24;13(5):e0198064. doi: 10.1371/journal.pone.0198064 (PMC5967717; doi:10.1371/journal.pone.0198064)
Supplement: S1 Table — (DOCX) [file pone.0198064.s001.docx]

**Supporting Information**

**S1:** Two-way analysis of variance data for behavioral phenotypes

| **12 weeks body weight** | P value summary | F(DFn, DFd) |
| --- | --- | --- |
| Perinatal Treatment | 0.6 | F (2, 42) = 0.51 |
| Sex | 0.0001 | F (1, 42) = 770 |
| Sex X perinatal treatment interaction | 0.95 | F (2, 42) = 0.05 |
|  |  |  |
| **Locomotor activity** |  |  |
| Perinatal Treatment | 0.35 | F (2, 44) = 1.09 |
| Sex | 0.08 | F (1, 44) = 3.19 |
| Sex X perinatal treatment interaction | 0.43 | F (2, 44) = 0.85 |
|  |  |  |
| **Elevated plus maze (% time in open arms)** |  |  |
| Perinatal Treatment | 0.95 | F (2, 27) = 0.06 |
| Sex | 0.07 | F (1, 27) = 3.92 |
| Sex X perinatal treatment interaction | 0.58 | F (2, 27) = 0.56 |
|  |  |  |
| **Elevated plus maze (number of entry to open arm)** |  |  |
| Perinatal Treatment | 0.94 | F (2, 27) = 0.06 |
| Sex | 0.09 | F (1, 27) = 3.16 |
| Sex X perinatal treatment interaction | 0.98 | F (2, 27) = 0.02 |
|  |  |  |
| **Spatial working memory (% spontaneous alternation)** |  |  |
| Perinatal Treatment | 0.005 | F (2, 32) = 5.01 |
| Sex | 0.56 | F (1, 32) = 0.34 |
| Sex X perinatal treatment interaction | 0.013 | F (2, 32) = 6.37 |
|  |  |  |
| **Object based attention (% recognition index)** |  |  |
| Perinatal Treatment | 0.04 | F (2, 30) = 3.41 |
| Sex | 0.14 | F (1, 30) = 2.35 |
| Sex X perinatal treatment interaction | 0.04 | F (2, 30) = 3.39 |
|  |  |  |
| **Cliff Avoidance Reflex (Latency to first fall)** |  |  |
| Perinatal Treatment | 0.99 | F (2, 30) = 0.003 |
| Sex | 0.08 | F (1, 30) = 3.35 |
| Sex X perinatal treatment interaction | 0.63 | F (2, 30) = 0.47 |
