# Supplementary material for: Attention and working memory deficits in a perinatal nicotine exposure mouse model
Source: PLoS One. 2018 May 24;13(5):e0198064. doi: 10.1371/journal.pone.0198064 (PMC5967717; doi:10.1371/journal.pone.0198064)
Supplement: S2 Table — (DOCX) [file pone.0198064.s002.docx]

**Supporting Information**

**S2:** Bonferroni post hoc multiple comparison data for behavioral phenotypes

| **12 weeks body weight** | P value summary | t | df |
| --- | --- | --- | --- |
| Male - Female |  |  |  |
| W | 0.0001 | 15.86 | 42 |
| S | 0.0001 | 16.29 | 42 |
| N + S | 0.0001 | 15.94 | 42 |
|  |  |  |  |
| **Spatial working memory (% alternation)** |  |  |  |
| Male |  |  |  |
| W vs. S | 0.99 | 0.05 | 32 |
| W vs. N + S | 0.0004 | 4.38 | 32 |
| S vs. N + S | 0.0009 | 4.05 | 32 |
|  |  |  |  |
| Female |  |  |  |
| W vs. S | 0.99 | 0.23 | 32 |
| W vs. N + S | 0.99 | 0.35 | 32 |
| S vs. N + S | 0.99 | 0.12 | 32 |
|  |  |  |  |
| **Object based attention** |  |  |  |
| Male |  |  |  |
| W vs. S | 0.99 | 0.12 | 30 |
| W vs. N + S | 0.014 | 3.05 | 30 |
| S vs. N + S | 0.011 | 3.17 | 30 |

W = Perinatal treatment with plain drinking water; S = perinatal treatment with 2% saccharin in drinking water; N+S = Perinatal treatment with 100 µg/ml nicotine + 2% saccharin in drinking water.
